# Multiple Arterial Thrombosis in a COVID Patient With No Known Comorbidities With Mild Elevation of D-Dimer

**DOI:** 10.7759/cureus.13207

**Published:** 2021-02-07

**Authors:** Nader Mekheal, Sherif Roman, Patrick Michael

**Affiliations:** 1 Internal Medicine, St. Joseph's University Medical Center, Paterson, USA

**Keywords:** covid 19, arterial thrombosis, hypercoagulability, d-dimer, multiple arterial thrombosis, coronavirus, limb ischemia, stroke, sars-cov-2

## Abstract

Since the coronavirus (COVID-19) pandemic started, new challenges have emerged regarding the management of coronavirus-infected patients. One of the most known devastating complications associated with COVID-19 is hypercoagulability. This can lead to severe disability or even death, especially in critically ill patients with known chronic comorbidities such as hypertension (HTN) and diabetes. D-dimer and clinical condition are among the most important tools currently used by clinicians to guide therapy and anticoagulation prophylaxis. Here we present a case of a COVID-19-infected patient with no known comorbidities and mild elevation in initial D-dimer level who had a rapid deterioration ultimately leading to death within weeks of admission.

## Introduction

Since the beginning of coronavirus (COVID-19) pandemic in December of 2019, there has been an increase in reported cases of COVID patients with various arterial and venous thrombosis resulting in severe complications such as limb ischemia, pulmonary embolism, stroke, or even death [1,2]. The majority of these complications happens in critically ill patients with several comorbidities in their medical history and are associated with elevated D-dimer on presentation regardless of chemoprophylaxis used during their hospitalizations [3]. There have been several studies in the literature theorizing the possible mechanism responsible for these complications. One of the most proposed hypotheses is that the virus leads to activation of systemic inflammatory state associated with cytokine storm and complement activation [1,2]. In this paper, we present a case of a COVID-19 patient with no known previous comorbidities and no significant rise of D-dimer on admission complicated with multiple arterial thrombosis with rapid deterioration of her course over days from admission.

## Case presentation

A 60-year-old female with no past medical history presented to the emergency department (ED) with a chief complaint of fever for nine days that has not been resolving. She also reported five episodes of loose stool x 2 days. Fever and diarrhea were associated with sweating, chills, nausea, and vomiting; however, no significant respiratory symptoms were reported initially. On admission, her oxygen saturation was 87% on room air. Labs were significant for ferritin 13360, lactate dehydrogenase (LDH) 403, D-dimer of 500 ng/ml, elevated inflammatory marker, and positive COVID nasal swab. Chest x-ray showed increased pulmonary parenchymal opacification over middle and lower lung zones. Initially, the patient was placed on nasal cannula; however, on day 2 her condition started to decompensate very rapidly. She was intubated and transferred to intensive care unit (ICU) for management of acute respiratory failure secondary to COVID-19 pneumonia. The patient was started on prophylactic dose of enoxaparin as initial D-dimer was mildly elevated.

**Figure 1:**
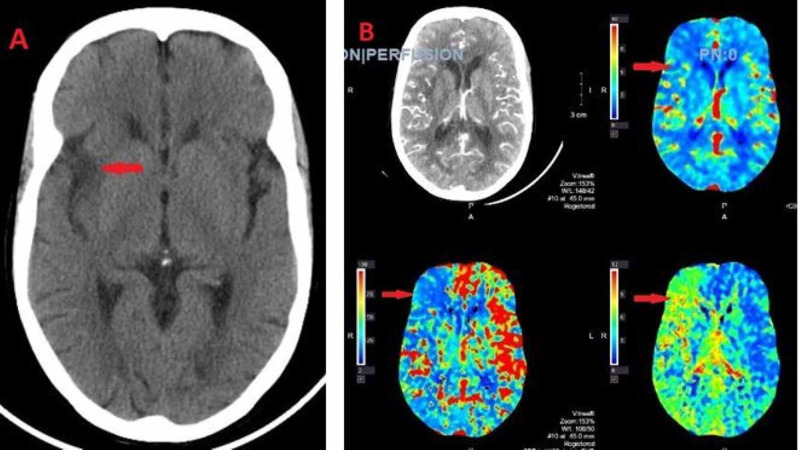
(A) Computed tomography (CT) head with no contrast showing lucency with loss of gray-white differentiation along the right insular cortex and subinsular region compatible with an acute infarct. (B) CT angiogram of the head showing perfusion defect in the right insular cortex and right frontal lobe suggesting a completed core infarct.

**Figure 2:**
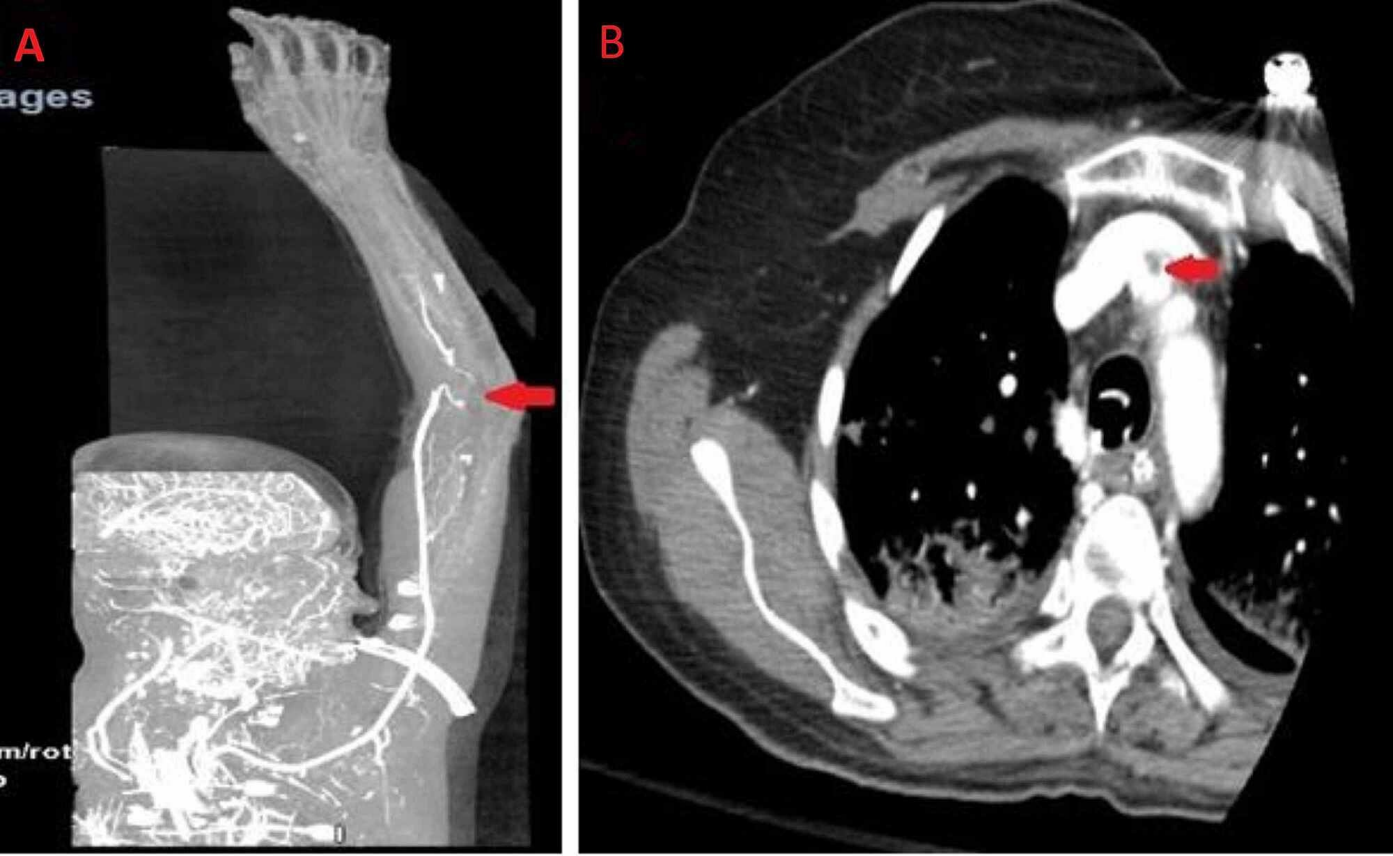
Computed tomography (CT) angiogram of right upper extremity showing occlusion of the most distal aspect of the right brachial artery at the bifurcation with no significant flow seen in the radial and ulnar arteries of the forearm (A) and small thrombus in the right innominate artery (B).

On day 5, it was noted that the patient had a new-onset left facial droop. Computed tomography (CT) of head was done, which showed the right insular and subinsular hypodensity concerning an acute right middle cerebral artery (MCA) infarct (Figure 1). Also, on the same day, the right hand was felt to be cold. Bedside Doppler revealed no radial pulse. CT angiogram showed occlusion of the most distal aspect of the right brachial artery at the bifurcation with no significant flow seen in the radial and ulnar arteries of the forearm, and a small amount of thrombus was seen within the origin of the right innominate artery (Figure 2). The decision was taken not to proceed with tissue plasminogen activator (TPA) due to the possibility of hemorrhagic conversion of MCA stroke. Two-dimensional (2D) transthoracic echocardiography was done, which was negative for any thrombus formation in the left atrium and ventricle.

On day 6, the patient had an embolectomy of the right brachial, radial, and ulnar artery with successful reperfusion of the right arm. She was started on heparin drip; however, her respiratory status continued to deteriorate complicated by sepsis requiring antibiotics and vasopressors until she eventually transitioned to comfort care and expired on day 26.

## Discussion

Even though critical ICU patients are at increased risk for hypercoagulable state commonly due to immobilization, multiple studies have been suggesting that SARS-CoV2 can directly cause hypercoagulability through increased inflammatory response manifested by increased C-reactive protein, LDH, ferritin, interleukin-6, and fibrinogen. One of the proposed mechanisms in which SARS-CoV2 can initiate such an inflammatory response is by directly invading endothelial cells in pulmonary alveoli through angiotensin-converting enzyme 2 (ACE2) receptors, starting a cycle of inflammation and thrombosis [1,2]. Ackermann et al. examined the lungs of seven patients who died from COVID-19. They found a greater number of ACE2-positive endothelial cells with significant disruption of intercellular junctions and cell swelling, which further supports this theory [4].

There have been many reported COVID-19 cases with different thromboembolism complications; most of these cases, if not all, have known previous comorbidities. Etkin et al. studied 12,630 hospitalized patients with COVID-19 in which 49 patients with arterial thromboembolism were identified. Most of these patients were men over the age of 65 and had multiple comorbidities such as hypertension (HTN) and diabetes, unlike our patient, a female, aged less than 65 and had no known medical history. All these patients presented with elevated D-dimer with a majority (83%) greater than 1000 ng/ml [3]. Similarly, Lodigiani et al. reported a median D-dimer of 529 ng/ml on admission that trended up to 1494 ng/ml a week later in non-survivors [1,5]. On the alternative, our patient presented with D-dimer level of 500 ng/ml with follow-up levels persistently less than 1000 ng/ml until rapid deterioration of her case on day 5 with D-dimer level of 20,000 ng/ml, which raises the question of the utility of D-dimer in considering COVID-19 patients for anticoagulation and assessing the severity of the disease on presentation.

So far, literature has been limited regarding the criteria for anticoagulation in COVID-19 patients. Physicians are faced with the question of whether to continue the regular prophylactic dose of anticoagulation or switch these patients to intermediate dose and if continuing anticoagulation is needed post-discharge [6]. Tang et al. found that 28-day mortality of COVID-19 patients with sepsis-induced coagulopathy (SIC) score - which can be calculated using international ratio (INR), platelet count, and Sequential Organ Failure Assessment (SOFA) score - of ≥4 or D-dimer of >3000 ng/ml treated with heparin is lower than non-heparin users (40% vs 64.2%, p = 0.029); however, there is no statistically significant change in the 28-day mortality if these criteria were not met [7]. Currently, physicians use combinations of D-dimer and patient medical history in dictating anticoagulation management; however, data supporting these recommendations are limited.

## Conclusions

Based on recent literature, there is no doubt that critically ill patients with past known previous comorbidities such as diabetes, HTN, and coronary artery disease are at increased risk for arterial and venous thromboembolism complications. Younger patients with no past medical history should also be observed for such complications. Also, as presented in our case, D-dimer can help consider these patients for anticoagulation; however, it is not always predictive. Much more research is needed to assess the sensitivity of D-dimer and its significance in actually changing morbidity and mortality.
